# Schooling impacts on the overactive bladder diagnosis in women

**DOI:** 10.1590/S1677-5538.IBJU.2016.0575

**Published:** 2017

**Authors:** Larissa R. Ferreira, Monica O. Gameiro, Paulo R. Kawano, Hamilto A. Yamamoto, Rodrigo Guerra, Leonardo O. Reis, João L. Amaro

**Affiliations:** 1 Departamento de Urologia, Faculdade de Medicina de Botucatu, Universidade Estadual Paulista, Botucatu, Brasil;; 2 Serviço de Reabilitação Perineal, Faculdade de Medicina de Botucatu, Universidade Estadual de São Paulo, Botucatu, Brasil;; 3 Faculdade de Medicina de Campinas, Pontifícia Universidade Católica de Campinas - PUC Campinas, Campinas, Brasil

**Keywords:** Women, Urinary Bladder, Diagnosis

## Abstract

**Objective:**

To evaluate the overactive bladder (OAB) diagnosis using OAB-V8 and ICIQOAB questionnaires in women with different schooling and cultural levels.

**Materials and Methods:**

Three hundred and eighty six healthy women answered a clinical questionnaire filling out information about schooling, demographic and gynecological data. The OAB-V8 and ICIQ-OAB questionnaires were used to evaluate OAB diagnosis and symptoms; and the QS-F questionnaire, to determine the sexual function. All questionnaires were validated in Portuguese.

**Results:**

The mean age was 37.3 years-old. Regarding to schooling level, 23.1% had concluded primary education; 65.8%, secondary school; and 11.1% had higher education. Considering the OAB-V8 (score ≥8), 51.8% of evaluated women had OAB diagnosis. There was a positive linear correlation between the OAB-V8 and ICIQ-OAB questionnaires in its sections “a” (r=0.812, p<0.001) and “b” (r=759, p<0.001). There was a positive linear correlation between age and the amount of time used to answer the OAB-V8, ICIQ-OAB and QS-F questionnaires (p<0.001).

The ICIQ-OAB was the hardest to answer for all schooling levels when compared to the other questionnaires. Women who had concluded primary and secondary education significantly demanded more help to answer all questionnaires than those with higher education (p<0.05). Furthermore, women with higher education took significantly less time answering all questionnaires when compared to their less educated counterparts (primary and secondary schooling), since they were quicker to answer each individual question.

**Conclusion:**

Educational level and ageing had an impact on women response using different questionnaires for OAB and sexual function evaluations.

## INTRODUCTION

Overactive Bladder (OAB) is defined by the International Continence Society (ICS) as urinary urgency usually accompanied by an increase in urinary frequency and nocturia, with or without urinary incontinence, in the absence of other local diseases (1).

The prevalence of this condition is high, as well as its impact on quality of life. It is estimated that in 2018, approximately 546 million people will suffer of this problem (2). In southern Brazil, it is estimated that approximately 18% of the population present symptoms of overactive bladder (3).

The use of specific clinical questionnaires can be an important tool in the OAB evaluation and diagnosis. These questionnaires assess the symptoms severity, the discomfort degree, and the influence on quality of life (4). Additionally, different authors have proposed the use of specific questionnaires for the evaluation of sexual function in OAB patients (5).

The OAB-V8 is a simplified version adapted from the symptom Bother Scale of the overactive bladder questionnaires (OAB-q), validated to Portuguese that may be used in OAB diagnosis. It consists of eight questions with domains of 0 to 5 and OAB is considered a probable diagnosis when the score is equal to or higher than eight (6, 7).

Due to a lack of studies evaluating the agreement and accuracy between the questionnaires used in the OAB diagnosis, this study proposed to estimate the impact of OAB on sexual function, and the agreement between the OAB-V8 and ICIQ-OAB questionnaires in women, considering different social and economic status.

## MATERIALS AND METHODS

Three hundred eighty-six healthy women, aged 18 years or older, were included from three different centers: 129 participants from the Physiotherapy Service; 129 from the Rehabilitation Center and 128 from the Hospital Estadual. As the study was developed in three different rehabilitation centers, we estimated the proportion of responders for each of them, taking into account the amount of care in each service.

All interviewed participants were undergoing physiotherapeutic treatment for non-urological complains, most of them, as companions of patients who were followed in these centers. Women were simply approached in the waiting room of these physiotherapy centers while waiting for care.

All participants were informed about the research, and, if they agreed to participate, they signed the free informed consent approved by the Ethical Research Board. The women answered a clinical questionnaire, which filled out information about schooling, demographic and gynecological data. The OAB-V8 and ICIQ-OAB questionnaires were used to evaluate OAB diagnosis and symptoms; the QS-F questionnaire was used to evaluate sexual function. All questionnaires were validated in Portuguese.

The OAB-V8 questionnaire consists of eight questions, with domains range between 0 to 5, and if the result is equal to or greater than 8 there is a probable diagnosis of overactive bladder (6).

The ICIQ-OAB evaluates the OAB symptoms in the “a” questions, and quality of life in the “b” questions, and highest scores correspond to worse situation regarding to a particular disorder (8).

The QS-F consists of 10 questions that evaluate all stages related to sexual function in women; the higher the score was, more favorable the situation (9).

The participants were subsequently invited to answer three questions: 1- Which questionnaire did you find the hardest to answer?; 2- Did you need to ask for help answering them?; 3- Which questionnaire demanded the most time for you to answer, in terms of minutes? The response time of each questionnaire was always recorded by the same researcher (a physiotherapist) using a digital chronometer.

The categorization of the schooling level of participants was established according to the organization and structure of education established by the Brazilian Ministry of Education (MEC) considering the information provided by the respondents (10).

### Statistical analysis

The sample size was determined using casual participation of the subject to the survey, considering the probability of 50% that each person would accept or not to participate in the study, estimating 10% standard error and 5% significance level for the conclusions.

Considering that the study was developed in three different rehabilitation centers, we estimated a proportion of responders for each of them, taking into account the amount of care in each service. The total sample size was calculated as 384 (11).

For clinical and demographic data analysis, the nonparametric chi-square test was used (12). The measurement of the strength of the linear relationship between the different variables was performed by the Pearson Correlation Coefficient when the variables were quantitative, and the Godman's Test was applied to establish the contrasts between qualitative variables within multinomial populations.

The Bonferroni Method was used to evaluate the variance analysis for models of repeated measures in independent groups (13–15).

## RESULTS

Clinical and demographic data of the study population are summarized in Table-1. Associated comorbidities were observed in 25% of participants: hypertension (10.6%), hypothyroidism (3.6%), Diabetes mellitus (2.1%), and other diseases (8.7%).

**Table 1:** Clinical and demographic data of the study population.

Characteristics of the population studied					
Variable		Mean	Variable	Total	%
Age	37.3 (14.4)	**Skin color** *			
BMI	27.0 (5.6)		White	288	74.6
Pregnancies	1.6 (1.8)		Mulatto	69	17.9
Vaginal delivery	0.6 (1.3)		Yellow	9	2.2
C-sections	0.8 (1.1)		Black	20	5.2
		**Civil status** *			
			Married	196	50.8
			Separated/ divorced	24	6.2
			Widow	14	3.6
			Single	152	39.4
		**Education** *			
			Primary	89	23.1
			Secondary	254	65.8
			Higher	43	11.1

* Categorical variables of the population studied, divided into sample size and their respective percentage

In 51.8%, the symptoms score was higher than or equal to 8 using OAB-V8, representing probable OAB diagnosis (6).

Average scores questions on the sections “a” and “b” of the ICIQ-OAB questionnaire was 3.08±0.16, and 7.19±0.61, respectively.

There was a positive linear correlation between the OAB-V8 and OAB ICIQ-scores in both sections “a” (r=0.812, p <0.001), and “b” (r=0.759, p<0.001).

Average QS-F score was 61.48±1.5, demonstrating that sexual performance in the women varied from regular to good (9).

There was a positive linear correlation between age and response time in all questionnaires, suggesting that older women need longer time to answer these questionnaires (Table-2).

**Table 2:** Pearson linear correlation: age versus time to answer the questionnaires.

	Linear correlation	
Association	“r” Value	“p” Value
Age x Time OAB-V8	0.190	p<0.001
Age x Time ICIQ-OAB	0.281	p<0.001
Age x Time QS-F	0.291	p<0.001

There was a negative linear correlation between the OAB-V8 questionnaire as compared to the QS-F (r=-0.519, p=0.033).

ICIQ-OAB questionnaire was significantly more difficult to answer when compared to other ones in all level of education. However, there was no statistical difference among the different levels of education considering each questionnaire separately (Table-3).

**Table 3:** Number and percentage of women considering ease to answer the different questionnaires according to educational levels.

Education	OAB-V8	ICIQ-OAB	QS-F	Total
Primary	20 (42.6%) **aB**	6 (12.8%) **aA**	21(44.6%) **aB**	47 (100%)
Secondary	66 (41.0%) **aB**	38 (9.8%) **aA**	57(35.4%) **aB**	161(100%)
Higher	10 (55.6%) **aB**	2 (11.1%) **aA**	6 (33.3%) **aAB**	18 (100%)
**Total**	96 (100%)	46(100%)	84 (100%)	226(100%)

Different lower cases mean statistically significant differences among educational level in the same questionnaire.

Different upper cases mean statistically significant difference considering different questionnaires in the same educational level.

Women with higher educational levels needed less assistance to answer any of the questionnaires when compared to other educational levels. However, in higher level about 15% of participants required some degree of help during filling out of OAB-V8 (Table-4).

**Table 4:** Number and percentage of women who needed assistance to answer the questionnaires according to the level of education.

Education	OAB-V8	ICIQ-OAB	QS-F	Total
Primary	5 (38.4%) **bA**	4 (30.8%) **bA**	4 (30.8%) **bA**	13 (100%)
Secondary	33 (46.4%**) bA**	19 (26.8%) **bA**	19 (26.8%) **bA**	71 (100%)
Higher	3 (15.2%) **aB**	0 (0%**) aA**	0 (0%) **aA**	3 (100%)
**Total**	41 (100%)	23 (100%)	23 (100%)	87 (100%)

Different lower cases mean statistically significant differences among educational level in the same questionnaire.

Different upper cases mean statistically significant difference considering different questionnaires in the same educational level.

It was found a significantly shorter time to answer the questions in all questionnaires in higher education level when compared to other educational levels (Table-5). Similar results were observed when compared secondary and primary education levels (Table-5), demonstrating the importance of this parameter in the evaluation of patients using specific questionnaires.

**Table 5:** Response time (seconds) for each questionnaire according to education level.

Education	OAB-V8	ICIQ-OAB	QS-F
Primary	134.4±56.5 **c**	137.1±55.7 **c**	200.1±79.6 **c**
Secondary	107.6±40.0 **b**	107.2±35.8 **b**	146.6±46.3 **b**
Higher	89.7±34.7 **a**	91±28.5 **a**	123±40.6 **a**
**p value**	p<0.001	p<0.001	p<0.001

Different lower cases mean statistically significant differences different educational levels.

## DISCUSSION

According to AUA guidelines, OAB is not a disease; it is a symptom complex that generally is not a life-threatening condition. In this context, in pursuing a treatment plan the clinician should carefully weigh the potential benefit to the patient of a particular treatment against that treatment's risk for adverse events and its severity and reversibility. However, if after the assessment has been performed to exclude conditions requiring treatment and counseling, no treatment is an acceptable choice made by some patients and caregivers (16).

Once OAB is determined by subjective and objective symptoms, the patient's perspective is very important in the proper management of this important condition. The diagnosis of this clinical syndrome is based on detailed history, physical examination, and urine analysis. Additionally, OAB may negatively affect sexual activity and orgasm. Different authors have demonstrated decrease in the orgasm in these patients (5).

For this reason, several diagnostic tools evaluating patient reported outcomes are proposed in attempt to help diagnosis. In some patients, the voiding diary may be used, as well as the measures of post-void residual urine (4). More complex diagnostic procedures such as urodynamic study may be not necessary, and it is reserved only to specific cases such as in neurologic disease, high post-void residue or pharmacological treatment failure (17).

The ICIQ-OAB questionnaire has level “A” of evidence, and provides assessment of urinary symptoms such as frequency and urgency, and also measures their impact on quality of life. Bothersome related to OAB symptoms are worse when final questionnaires score is higher (8).

Some authors have reported that age, BMI, pregnancy, type of delivery, and ethnicity may influence the incidence of OAB diagnosed by validated questionnaires (18–20). Considering the World Health Organization (WHO) (21) classification, we noted that most of women in our study were white overweight mature adults.

In our population, the most common comorbidities were arterial hypertension and Diabetes mellitus (DM). Some authors had stated that these comorbidities and depression might be associated with OAB (22, 23).

We observed a probable OAB diagnosis in more than half of women using the OAB-V8 questionnaire. Davila et al. (24) reported similar results (51%) using the same questionnaire, and also questioned the reliability of isolated use of this instrument for OAB diagnosis. Thus, if we consider only the OAB-V8 questionnaire our outcomes may be overestimated.

Lapitan et al. (25) identified a global prevalence of OAB of 53.1% in an Asian female population, using a questionnaire specific to the region. Other studies report a prevalence ranging from 11.8% to 18.9% using different questionnaires (2, 26, 27). Some authors attribute this variation in prevalence rates to the different definitions and questionnaires used for the diagnosis of OAB (28). Scafuri et al. (29) stated that it is difficulty to select the adequate tools for assessment and diagnosis of OAB, impacting the divergences of OAB prevalence among different studies.

In Brazil, the questionnaires frequently used for OAB prevalence are not specific. So, using a specific questionnaire, we found a high prevalence of this disorder. It's important to remember that in our protocol we evaluated only female participants.

In their study, Teloken et al. concluded that overactive bladder is a highly prevalent condition, even in young populations (3). It affects both genders, yet it is more frequently observed in women (14.0% in men versus 23.2% in women; overall 18.9%). According to the author, the lack of standardization in the diagnosis and utilization of different criteria in scientific papers may hinder a comparative analysis.

Additionally, they applied a different strategy to evaluate their patients, once they use a questionnaire developed by the combination of questions from the King's Health Questionnaire validated for OAB syndrome, the AUA Symptoms Score and original questions.

Although our study has shown a possible overestimation in the OAB prevalence, there was a positive linear correlation between the OAB-V8 and ICIQ-OAB questionnaires, demonstrating that there was concordance in the OAB diagnosis and the answers about worsening on OAB symptoms and quality of life.

In the present study, the longer response time in all questionnaires observed in elderly women may reflect a reduced cognitive capacity resulting from the physiological aging process and mild cognitive decline, characterized by memory and attention deficits (30).

According to Vallet (31), some alterations in perception and cognition may occur during the process of physiological aging, such as reduced cognitive processing ability, reduced attention in the execution of some functions, as well as reduction in the ability of free recall and in episodic memory.

We observed that women had more difficulty in answering the ICIQ-OAB questionnaire in comparison to OAB-V8. This fact is in disagreement with other authors that have described the ICIQ-OAB as a single and objective questionnaire to assess the storage symptoms related to OAB (32, 33), suitable to use in different groups, young or elderly patients.

The QS-F questionnaire was specifically developed for the Brazilian population to evaluate the female sexual function. Actually, the studies available so far do not discuss or compare the response time with the level of education (9, 34). We observed a clear influence of the education level and the response time, demonstrating that women with higher level of education needed short time to answer these questionnaires. To our knowledge this is the first report in the current literature to address this question.

Our results showed that women who had only primary and secondary levels of education needed more assistance to answer the questionnaires. Some authors have reported that individuals with higher levels of education could solve more easily some problems and specific cognitive functions (35, 36). These facts justify why women with higher levels of education had shorter response time and less assistance to answer the questions in our study.

There was a negative linear correlation between the specific questionnaire for OAB symptoms and sexual function (SF), where higher scores for OAB-related symptoms had worst results in SF. This fact demonstrates the negative influence of OAB symptoms in sexual performance.

In conclusion, educational level and ageing had an impact on women response using different questionnaires for OAB and SF evaluations.
